# “When You Make a Movie, and You See Your Story There, You Can Hold It”: Qualitative Exploration of Collaborative Filmmaking as a Therapeutic Tool for Veterans

**DOI:** 10.3389/fpsyg.2018.01954

**Published:** 2018-10-16

**Authors:** Rivka Tuval-Mashiach, Benjamin W. Patton, Charles Drebing

**Affiliations:** ^1^Department of Psychology, Bar-Ilan University, Ramat Gan, Israel; ^2^Patton Veterans Project, New York, NY, United States; ^3^Bedford VA Medical Center, Edith Nourse Rogers Memorial Veterans Hospital, Bedford, MA, United States

**Keywords:** reintegration, veterans, filmmaking, digital storytelling, PTSD, qualitative research, narrative, art therapy

## Abstract

Despite the availability of effective treatments for coping with traumatic experiences, a large percentage of military veterans in need do not seek help. The “I Was There” model is a new filmmaking program which is a creative-expressive tool, developed to enable veterans to reflect on their experiences and jointly create short artistic films. These artistic films articulate, often metaphorically, aspects of the veterans’ service experiences, traumatic events, and reintegration challenges. The current study employed a qualitative methodology to explore participants’ subjective experience of the program. We interviewed 50 participants following the intervention, focusing specifically on their perceptions of the filmmaking process, the aspects they viewed as meaningful, and whether and how the process affected them. Most participants reported their experience as positive and empowering. Three overarching themes emerged as significant in describing the benefits of participation: Gaining a new sense of agency, regaining a sense of affiliation, and processing the trauma. The findings are illustrated and discussed within the context of narrative therapy, as is the potential of video-based therapy, especially regarding non-articulated, sensory traumatic memories, and for the process of (re)construction of the trauma narrative.

## Introduction

Reintegration after military service may be a challenging process for many veterans and service members. First, military service involves a unique context of exposure to stress and trauma. Often, it involves extended tours of deployment that exceed 1 year and are carried out in locations that are far from home and from the soldiers’ natural support systems; there is also ongoing exposure to the risk of death, injury, or sexual trauma (Bowling and Sherman, 2008; Richardson et al., 2010). Therefore, reintegration requires an extended process of adjustment. Factors that can complicate adjustment include long separations, the exigencies of leaving the military and seeking new jobs, family and relationship difficulties, the existence of injuries or disabling medical conditions, aggressive behavior, and substance use (Seal et al., 2009).

The challenges of social and familial reintegration are further exacerbated when the returning service member suffers from PTSD, traumatic brain injury (TBI), or other mental or physical health conditions, such as depressive symptoms, grief responses, or compromised physical health (Hoge et al., 2007). These symptoms may interfere with a veteran’s ability or willingness to directly communicate what he or she is experiencing to loved ones and/or mental health providers, further complicating reintegration and recovery. Over the past 15 years, about 2.5 million United States military personnel have served in Afghanistan and Iraq (Siegel and Davis, 2013). Around 75% of them reported multiple exposures during deployment (Tanielian and Jaycox, 2008). About 20% of them meet the criteria for PTSD (Peterson et al., 2011), and between 10 and 23% have had a deployment-related TBI (Sayer et al., 2015). There is evidence that the prevalence of reported mental health concerns and TBI increases as time since deployment increases (Polusny et al., 2011). When veterans cope with reintegration issues or stress related to their military service, the potential ripple effect can be enormous, affecting millions of other people at least indirectly (Richardson et al., 2010).

Despite the existence and availability of efficacious treatments, (e.g., prolonged exposure, cognitive processing therapy, medications), between 60 and 75% of distressed veterans do not seek treatment (Hoge et al., 2006). This finding may be explained by the stigma associated with treatment, institutional barriers (e.g., staff skill and sensitivity), and/or logistical barriers such as accessibility of service (Ouimette et al., 2011). Moreover, many veterans who do receive treatment often remain significantly symptomatic (Yehuda and Hoge, 2016). It may be that although traditional treatments for PTSD address some of the attendant symptoms of PTSD, they do not address the more complex issues associated with readjustment; for instance, veterans’ sense that they have changed and must somehow communicate to others that their deployment-related experiences have led to a profound transformation. The use of a powerful creative intervention can be helpful in facilitating this important process.

### Filmmaking and Video-Based Therapy

Video has been used as an element in therapy in recent years for different populations and in the treatment of various psychological issues, including post-traumatic stress disorder, or PTSD (Wedding and Niemiec, 2003; Gantt and Tinnin, 2007, 2009; Johnson and Alderson, 2008; Nanda et al., 2010). However, despite the growing use of its growing use of the video medium in various therapeutic settings, the field is still in the nascent stages of development; therefore, no consensus has yet been reached regarding what precisely constitutes video-based or filmmaking therapy or how it works to alleviate patients’ suffering (Johnson and Alderson, 2008). Because films can be used in different ways and because they serve various purposes in therapy, the field is still lacking a definitive nomenclature. For example, the term “video therapy” is currently used to describe several uses of video in therapy, such as watching films (cinematherapy), video-recording oneself in order to witness and reflect on one’s behavior, or making films (Cohen, 2013). Consequently, several overlapping definitions and uses of video therapy exist. In addition, evidence of the effectiveness of using the video medium in therapy is only slowly beginning to emerge (Malchiodi, 2015). Lastly, there is a debate as to whether video- and filmmaking therapy should be considered part of art therapy or whether it should be considered its own, separate therapy. That is to say, although some scholars (e.g., Malchiodi) view video-based therapy as falling under the category of art therapy, others suggest that it differs in some essential ways from other creative arts therapies. Cohen and Orr (2015) suggest that video-based therapy shares certain common aspects with creative arts therapies including projection (where the video can be used to engage with difficult materials), the use of one’s imagination, the enactment of bodily sensations and emotions, and editing, which involves creativity and sense-making (Cohen and Orr, 2015). In contrast, Johnson (2015) differentiates therapeutic filmmaking from creative arts therapies in its focus on the product, which is usually not the focus of the latter. Another significant difference Johnson describes is the film’s unique relationship with the dimension of time. That is, filmmaking can be seen as being “multiply therapeutic,” as it is characterized by both the benefits of the timeless arts (such as sculpture, and drawing) as well as those of the time-based arts (such as drama or dance).

Because the field is still developing, we consider it important to offer our theoretical understanding of the way we use the video medium as a therapeutic tool, as well as the practical ways in which we use it. We view the use of films and video in therapy as operating on a continuum between being very artistic and creative (i.e., and therefore falling under the rubric of the creative arts therapies) and being a form of simple, non-stylized “digital storytelling” (for a detailed description of the continuum, see Barak and Tuval-Mashiach, unpublished). The use of video in our model is positioned on the side of the artistic pole. The main theory we base our work on is the narrative approach (Angus and McLeod, 2004; Bruner, 2004; Schiff, 2017).

### Theoretical Rationale for the “I Was There” (IWT) Film Program Model

According to the narrative approach to therapy, when a traumatic event occurs, it challenges one’s identity in profound ways (Herman, 1992; Crossley, 2000; Tuval-Mashiach et al., 2004). It is often quite difficult to integrate the experience into one’s autobiography without taking stock of how this event affects one’s sense of standing in the world. Survivors may be struggling with issues of agency and control over their lives, changes in how they view the world and/or themselves, and their futures (Tuval-Mashiach and Patton, 2015). Narrative, as an organizing concept, is “the framework that is needed in the psychotherapy practice, in order to *retrieve* and *convey* the life experiences that have been stored in the body and converted into symptoms: the damage must be recovered, converted into language, related. The stories must be retrieved from somewhere far away, sometimes from far beyond the frontiers of consciousness. A person needs to be able to put the damage that has been caused into words, to “narrate” it” (Olthof, 2018, p. XXIX).

In line with the narrative theory, research has shown that being able to share aspects of one’s trauma narrative is therapeutic, as the narration of trauma gives rise to has many physical and psychological health benefits, including a decrease in PTSD symptom levels (Pennebaker and Seagal, 1999; O’Kearney and Perrott, 2006; Mowatt and Bennett, 2011; Castillo et al., 2012). Recently it has been found that even when the traumatic narrative is only being written, the therapeutic effect is not less positive than other evidence based treatments for PTSD (Sloan et al., 2018). On the basis of the narrative approach and in line with Malchiodi (2015) we view filmmaking much the way we view storytelling. The production of films is essentially a narrative process, wherein directors and their teams use digital video and audio technology to convey their messages. However, filmmaking differs from oral narration in several important ways that make it potentially more attractive and engaging for military veterans, especially younger ones. First, contrary to writing and talking, which are solitary activities, video production is inherently collaborative: It is very difficult to make a film alone. Second, PTSD is primarily a multisensorial disorder in that the memories of traumatic experiences are vivid, are auditory and visual, and often cannot be expressed in words. The more traditional verbal approach to therapy can be enhanced by adding a multisensory and more synoptic visual means of creating a narrative. Lastly, filmmaking involves symbolizing in a more explicit ways than verbal narration does (Stepakoff, 2007).

### The “I Was There” (IWT) Film Program

“I was There” film intervention model was developed by Benjamin Patton as a practical implementation of video-based intervention for veterans coping with reintegration issues and service-related trauma (Tuval-Mashiach and Patton, 2015). Rather than replace existing therapeutic modalities, approaches, or professional creative arts therapies services available to combat veterans available to combat veterans, (e.g., Gantt and Tinnin, 2007, 2009), its aim has been to serve as a new approach, which is theoretically based on combining the therapeutic value of narrative reconstruction with the artistic and expressive power of the filmmaking medium.

#### Procedure

The program consists of four half-day sessions, during which a team of professional video editors/filmmakers supervise the sessions, with up to 15 participants per session. A mental health provider is onsite throughout every workshop. Participation is voluntary, and veterans can choose to drop out of the program at any time. The only criterion that must be fulfilled in order for a participant to attend an IWT program is that he/she is considered to be a service member and that he/she has been exposed to military-related stress or trauma. Participants fill in several questionnaires both before and after the workshop (e.g., demographic information, PTSD assessment, and measures of well-being). Participants are interviewed at the end of the workshop.

The filmmaking process is similar to the five-stage process suggested by Johnson (2015), which includes the following stages:

(1) Development (developing and writing the story that the filmmaker wants to tell).
(2) Pre-production (i.e., preparing to shoot the story; this stage involves storyboarding, creating shot lists, etc).
(3) Production (the process of filming).
(4) Post-production (i.e., editing and adding pictures, effects, and sounds).
(5) Exhibition/Distribution (presenting or distributing the film to others).

#### The Program

On the first day participants learn basic directing and filmmaking skills, and conduct several filming exercises, to familiarize themselves with the video camera. On the second day, they are invited to share a topic they view as important and/or challenging in their coping with their condition, and a group discussion is encouraged, where participants listen to others’ stories and are given the opportunity to relate to them. Following this session, participants break up into small groups (3–4 people per group), coming together with others who share similar ideas and issues. During the second part of Day 2, and during Day 3, they choose a shared theme around which they will make their short film. Participants write the script, act, and shoot, all during the course of the third day, and on Day 4, in the post-production stage, they edit their films, add effects and sounds, and finalize their films. The last part of the fourth and final day is the screening of the films. The films are screened before an audience consisting of the other participants and the guests they choose to invite. During the screening, they present their films, and discuss them. Throughout the 4 days, the professional team facilitates the process of the filmmaking and assists with the technical aspects of production. The films usually involve the use of metaphors, symbols, and performance. The model for this intervention is based on three basic principles – listening, collaboration, and empowerment. The underlying rationale is that people who were exposed to trauma – whether they develop posttraumatic stress disorder (PTSD) or not – may still cope with other issues (van der Kolk et al., 2005). These include the individuals’ feelings of helplessness and despair, difficulties in making sense of the traumatic events that befell them and/or integrating them into their narratives and life plans, difficulties in communicating with others, and as a result, experiencing social loneliness and isolation (Shay, 2010; Stein and Tuval-Mashiach, 2015a,b).

As of mid-2017, nearly 40 IWT programs have been held in the United States, and several have been held in Israel as well, with the participation of more than 500 veterans; as a result, more than 300 films have been produced. The participant attrition rate has been under five percent. In terms of design and style, the films produced to date have represented a variety of media and genres, including spoken word/poetry, stop-action animation, music videos, public service announcements, marketing/promotion, comedies, documentaries, and narrative fiction. In terms of content, the films produced at the IWT film programs generally center on themes relevant to the experiences of the participating veterans, including combat, physical or psychological injury, personal loss, stigma, suicide, military sexual trauma, domestic abuse, medical retirement, transition to civilian life, parenthood and family, and spirituality. A sample of selected films produced in the programs is available here: http://iwastherefilms.org/featured-films/. Currently, the impact of the programs in alleviating PTSD symptoms and improving the participants’ well-being, and their effectiveness as an engagement tool for veterans, are being evaluated in randomized controlled trials, which are being conducted in collaboration with the U.S. Department of Veterans Affairs (VA). Preliminary findings show a steady and significant PTSD symptom level decline of 20% on average at the end of participation, an effect that has been found to be stable across a time period of 60 days (Tuval-Mashiach et al., unpublished).

### The Current Study

This article’s goal is twofold: First, we sought to qualitatively explore the experiences of program participants and to discover what it was that they perceived as meaningful and effective. Second, we wished to uncover the various mechanisms of change elicited by video therapy, given that – as previously mentioned – they are still for the most part unknown. As such, we aimed to delve into the participants’ experiences, to use their subjective perspectives to help us gain a better understanding of the potential mechanisms of change underlying this approach.

## Materials and Methods

### Sample

Fifty participants (40 men) were selected for the purpose of this study, out of a total of 500 veterans who have participated in the IWT programs. We used purposive sampling (Polkinghorne, 2005) with the aim of having participants from 10 different programs (five from each program), in order to represent the larger sample. The participants’ ages ranged from 19 to 65 (*M* = 31.6). Of the participants, 23 were married (46%), 18 were single (36%), and the remainder were divorced or separated (18%). About 60% were parents to at least one child. The participants’ military experience varied; the majority of them served in the United States Army (90%), with one having served in the Air Force, two in the Navy, six in the National Guard or Reserves, and one in the Marine Corps. In terms of deployment history, 10 participants (20%) had never deployed to a war zone, 17 were deployed once (35%), and the remaining 22 (44%) were deployed two times or more. About 30% reported being diagnosed with PTSD by a mental health professional.

### Study Design and Interview Protocol

Interviews were conducted on the last day of the program, in a quiet room on the workshop site. Most of the interviews were carried out by the two first authors, both of whom are psychologists, and are trained in interview research. The interviewers adhered to a general interview protocol while also maintaining flexibility. The aim of the interview was to learn about the experience of the program participants, focusing specifically on their perceptions of the filmmaking process, the aspects that they viewed as meaningful, and whether and how the process affected them. The first question was an open question, inviting participants to talk about what it was like for them to be part of the program. They were then asked several specific questions regarding what their expectations of the program had been, what they had hoped to get from it, about their previous therapeutic experience, what they thought about the use of video and about the filmmaking process, and what impact the process had made on them. The length of the interviews ranged from 10–20 min, and all interviews were video-recorded. For the current analysis, the first author watched all 50 interviews, and coded them for themes.

### Analysis

Interviews were analyzed for categorical content analysis on the basis of the model of narrative analysis proposed by Lieblich et al. (1998). After familiarizing ourselves with the interviews, the analysis consisted of generating initial codes by deconstructing the text into identifiable units of meaning. We combined confirmatory and exploratory approaches (Patton, 2005) in an attempt to extract meanings and recurring themes. Namely, the two first authors had identified existing categories derived from the literature (e.g., curative factors in arts therapies), and were prepared to generate new codes and themes that would emerge from the data in a bottom-up process. The next stage consisted of our search for themes and categories that would organize around the emerging codes, while we also sorted additional codes into those preexisting categories. The last step of the analysis consisted of reviewing the emerging themes and scrutinizing them for consistency.

### Ethical Considerations

All of the participants underwent a screening interview prior to the testimonial process in order to assess the likelihood of reactivation of traumatic experiences. In addition, with the aim of insuring confidentiality, all of the participants’ names were changed (Josselson, 2007). At the first program session, the program leader emphasized that anyone who was not interested in completing the program was welcome to leave at any time. After completion of the program, participants were given their films, and were once again told that they could decide whether or not they wished to share their films and make them available to the public. Only participants who signed a consent form permitting the submission of their interviews for research purposes were included. A mental health provider was on hand throughout each workshop session, in case a participant experienced anxiety and required immediate mental health support during a session.

## Results

The analysis revealed three overarching themes: Regaining a sense of agency, regaining a sense of affiliation, and processing the trauma. Although within the narratives themselves there were interrelated aspects of these three themes, here, for the sake of clarification, we present them as separate.

### Regaining a Sense of Agency

According to several trauma theories, (Herman, 1992) trauma by its nature ruptures one’s sense of control over one’s life. The traumatic event, which is often unexpected and uncontrolled, may lead to feelings of helplessness, victimization, and a sense of compromised agency. Based on this conceptualization, our model aims at enabling participants to regain a sense of agency and control over their behavior and choices, within the process. Therefore, a core principal of the model is that veterans are free to choose their level of participation in each of the program stages, and although they are encouraged to join the discussions, or the film production, they are also free to choose their level of involvement, what and how much to share about their trauma, the group with which they prefer to work, and their role in the film production team.

From the interviews, it appeared that participants enjoyed the ability to choose the right level of involvement and exposure for them. Most participants referred to this aspect spontaneously as one of the components they perceived as most meaningful and unique about the intervention. We termed this overarching theme *regaining a sense of agency* (rather than control), because the experiences that the men and women described dealt with more than recovering control. They were related to recovering a sense of being one’s own source of action and initiative. The sub-themes that were related to this notion were: *Feeling safe; viewing the role of the tutors as facilitators rather than as guides of the process;* and *freedom to reveal one’s story at one’s own pace*.

#### Feeling Safe

Promoting a sense of safety is common in most arts therapies and is often the focus in the early stages of therapy (Redfern, 2014). *Feeling safe* was mentioned in most of the narratives as one of the most important elements for the participants. Interestingly, because the IWT model is not built on a structured set of instructions, the beginning phases of the intervention were experienced as vague and without clear rules; participants were therefore unsure of what to expect, and that led to feelings of uneasiness. However, the moment participants understood that they were being invited to “hold the reins,” they felt motivated to proactively engage in the process; this moment seemed to serve as a turning point for them.

Tom, a 24 year old veteran said:

When I came, I expected I would have to talk about my PTSD, well, F^∗∗∗^. I don’t want to do that, it’s not my ideal goal of reliving things that I did so much to finally overcome and try to bring all that up. It wasn’t my ideal situation. But actually, hearing what the program is about, that it’s on any issue that you feel like needs to be expressed, I said, ok, well, that’s cool. I think it’s a better overview, not just sitting and focusing on one specific thing, because not everyone does experience PTSD, or experiences it in the same way. And it was better than I expected. It was completely better.

Several participants said they felt safe due to the non-judgmental atmosphere, and to the feeling that everyone was welcome. John described how the structure of the intervention allowed him to feel he could relax, open up, and be more engaged:

It was a different experience than many types of clinical settings where I’ve been. And it’s different than other group settings, where you have to talk about your feelings, and you have to explain why you’re even here… In other settings I felt very reserved, in a lot of the appointments that I’ve been at, where you have a 40-min window, and you never really get a chance to really talk, you can’t complete a thought. Here, the invitation, the doors are literally open. And there’s something about it that says: have at it, explore, feel free to relax, feel free to explore, and you sit back and say- well, I’ll take you up on that.

The sense of safety, which was created in the first stages of the intervention, enabled participants to feel in control throughout the whole process, even when it came time to discuss their own trauma. David, who was severely injured by a terrorist bombing said: “I can relive this nightmare, but in a safe environment. I’m not gonna… I’m in control. I can stop whenever I want.”

#### Role of the Professionals

As part of the freedom to choose their own level and type of engagement, participants noted the different role played by the group instructors in this intervention vs. other therapists they had worked with in previous therapeutic settings. Many mentioned that the instructors were there to “think together with us,” whereas others saw them as assisting the veterans in translating their ideas from an abstract place to a more concrete one, in the form of digital media. They helped them formulate their ideas, and offered editing suggestions, etc. We conclude this section with Amanda’s words about validation:

…. It gives me hope, that there is the ability for validating yourself and incorporating friends and family, not to shut everybody out, dealing with it yourself. You come to an environment like this where you can ask for help, and help can be available, and all the resources are there at your fingertips. So it kind of reaffirmed that, at the end of each day that I was here, I kind of had a lighter step, and a little more of a positive outlook. I feel like I sit upright.

#### The Ability to Regulate Exposure

Many participants reflected on their choices regarding their filmmaking process. Their descriptions showed that it was important for them to actively choose how much of their trauma – and which aspects of it – they wished to share. We illustrate this point through Beth’s story. Beth, who is a service member, was exposed to extremely difficult material on 9/11 as part of her work-related duties. Since that time, for the following 16 years, she never talked about what had happened. Beth decided to “give the program a shot,” and interestingly, decided *not* to join a group of other 9/11 female veterans, who planned to make a film on 9/11. Instead, she said:

I came to work, to get away from the girls who were in 9/11. I didn’t want to go there. I wanted to work with somebody, that it could be in there, but on a positive level. I didn’t want to relive the whole day, which was what the other girls were doing. So I joined someone who had a totally different idea, but putting my input somewhere there, that would be more effective for me. And it was.

This self-chosen exposure seemed to be right for Beth, as it enabled her to break out of the cycle of avoidance in which she had previously been trapped. When she shared her feelings about her trauma with the others, it occurred to her that 9/11 had been a happy day in her family prior to the terrorist attack, because of her child’s birthday. Since then, “the terrorists had taken over” that date. For her, the act of creating the film, and symbolically changing the story to a story that ends with hope, was transformative. Later, she was able to watch it without arousal:

I think a lot of people can benefit from it. Because I am very stubborn and I’m very close-minded to this whole idea of going back, and re-living any kind of grief. I don’t go there. I don’t touch it. It’s like taboo for me. So for you guys, to be able to help me with it, I think you can help a lot of other people. And I watched my film over and over, and I don’t have a problem with that.

### Regaining a Sense of Affiliation

When describing the challenges they faced, most veterans described feelings of loneliness and detachment. As is well documented in the literature (Stein and Tuval-Mashiach, 2015a,b), loneliness, alienation, detachment, and difficulties in sharing are part of the post-traumatic experience, and have been found to be related to distress and to dropout from therapy. From the interviews, it appeared that the intervention format, which emphasizes collaboration, eased feelings of loneliness and enhanced a sense of affiliation. Three sub-themes emerged in relation to this topic: *feeling understood, a willingness to share with others, and a sense of belonging*.

#### Feeling Understood

The majority of participants stated that they didn’t usually like to talk about their trauma-related memories and issues, for different reasons. Jane described how she “bottled up” all her emotions because she didn’t realize how severe her situation was. Jeff said he felt others wouldn’t be able to understand him, so he stopped trying. Others avoided talking about their memories because they were too painful, or because they wanted to protect the people closest to them. Several participants said they never shared anything and kept their memories to themselves, and these participants were especially reluctant to share at the beginning of the intervention. After the intervention began, however, all the participants said that they liked to share and that they perceived the collaboration component of the model, which encouraged engaging with other veterans, as the catalyst for their ability to open up. As can be seen from the examples we chose, feeling understood and being willing to share are intertwined. Each one seems to feed the other in the following manner: As the ability and wish to share grows, and an individual feels more and more understood, his/her feelings of loneliness decrease.

Jack, who after losing his friends during a mission left home to live abroad for many years, hoping to escape from the memories, said:

On the first day I came, I wasn’t so comfortable. On the second day, (pauses) it was like coming home (smiles). It was… I’ll tell you. I was assigned to a group with three men I had never met before. And there was a list of topics to discuss, and we were asked: what topics do you identify with? I felt: well, nothing clicks for me. I said: Well, I said to myself, if M & N (other veterans) want to make the film about whatever, I don’t mind, I’ll join them, it doesn’t really matter. We didn’t have anything in common. But 5 min after we started talking, I felt that I had so much in common with them, like with no other person I’ve met in my life before.

Jack carried a heavy burden of guilt and helplessness over his trauma, which he was ashamed to share; additionally, over time, as the years went by, he had felt increasingly isolated with his secret. During the intervention, the revelation that other veterans had experienced similar feelings, despite the different circumstances, was transformative and healing for him:

Everything that I experience, all the hard emotions – it’s what THEY feel too (smiles). I can’t stop thinking about it. For the last couple days, I can’t seem to stop talking about this feeling – I didn’t want the day to end. Suddenly I can talk. I have never shared anything, I never talked. And then, not only is it possible to talk about it, it’s ok to laugh at it…

Beth described why she felt it was important to work with other veterans:

It’s almost like, two recovering people. In a program, like, of addiction. Only one addict can know another addict. Only one alcoholic can understand another alcoholic. That’s how the program actually works. Cause if a regular person came and talked to them about their problem, they probably wouldn’t even listen to him, cause they don’t feel you relate. This is the same thing with PTSD. For me, this is my opinion. That if I’m talking to someone who understands how I’m feeling, it’s. It’s lighter. The load. It feels… it’s, I’m recovering at that moment. That I’m talking to someone who understands.

#### Willingness to Share

As with Jack and Beth, many participants emphasized that being with others who were like them – people who went through similar challenges and could understand them and their experiences – created an atmosphere where they felt safe and accepted without judgment. This experience was, for many, unexpected and surprising, and it promoted *openness, and a willingness to share*. As Dan said: “Each one has his own story, but how you react, how you feel, the problems you all go through are very similar…you have that immediate connection.”

Joan said:

Working with the PTSD group all week, hearing other people talking about their histories, learning about trauma. I had built trust with the people that were in the group. The day had something there. There was something with them, there was some kind of hope there.

#### Feelings of Belonging

The growing feeling of trust and viewing the other group members as capable of relating to their stories, made it easier for participants to share, and later to form groups to work on the films. Given that each group created only one film, the group members had to decide among themselves the subject of this film, and how it could best represent aspects of all of the group members’ experiences. The joint project of the filmmaking enhanced the *feelings of belonging*, and of being part of a group. Several participants mentioned that it felt like “being in the unit again,” a time when they had been part of something, all united toward achieving a specific purpose.

Cumulatively, these three themes together comprise what we term *regaining a sense of affiliation.* This sense was expressed both at the level of connections with the other participants *in* the intervention group, as well as with others, *outside* the group. This point is worth noting, because it is indicative of the film’s potential to encourage communication about the trauma with the veterans’ family members and friends. As an example of this idea, Tom said: “It is giving me the opportunity not only to help myself, but to help people understand me better.” Others mentioned that their film might be of help to other veterans who are struggling with similar issues, and might show them that hope exists. John said:

When I had the chance to show my film to my wife, the first thing she said was, simply, “I finally get it.” She could finally come to terms with, and understand, what I was going through that I couldn’t put into words.

Diane said, after being asked if she wanted to share her film:

At first, it was just for me, personally. But after seeing the whole make of it, now, when it actually came out, the music, everything just fit together, I’m actually pretty impressed, how me and my partners had put it together, so yes- friends, family, whoever. Whoever wants to see it.

The last theme refers to the unique aspects of filmmaking in the context of trauma. We termed this theme *processing the trauma*, and it relates to what participants saw as the inherent advantages of the filmmaking process.

### Processing the Trauma

Participants were asked to reflect on the process of the filmmaking itself, and to describe what they liked about it. Several themes which are unique to the IWT model emerged: *reconstructing the trauma narrative, distancing, and sense making.* In what follows, we describe these themes.

#### Reconstructing the Trauma Narrative

Filmmaking requires a plot, and the creation of a film requires the construction of a narrative. During the process of planning their films, participants were encouraged to use their imagination, think about their target, and construct their stories as they wished. Participants chose how to relate to their trauma, and whether to *mirror* a concrete aspect of their story, represent it in a *symbolic* way, or *transform* the narrative, in order to convey their message. David, whose group film represented a transformation of the trauma narrative, from negative to positive, said:

I can change the narrative, you know, the word “crisis” in Greek means crossroads and yes, you can go down, but you can also go up. I can feel like the victim of the guy who threw a grenade at me, and disabled me, but here, I can also feel that I won. I’m alive, I help others.

Joan, whose group dealt with the trauma symbolically, described her group’s film:

I knew exactly what I wanted to do, I wanted to let go of the rape, the shame, and so we came together, we started throwing out ideas, and the concept came up, of throwing the boxes (each representing a heavy negative emotion – *the authors*) and letting them go, being able to destroy all the bad emotions and stuff.

Another participant, Bill, described how making the film enabled him to go back to his trauma, and to process it differently. He spoke of the theme of *distancing* as a central component of the model. In his group, someone else was playing parts of his own story, and that “outsourcing” was experienced as a transformative experience for him: “It almost takes me to a place of being in the third person. Where I’m looking at myself from the outside. In this video. It has given me insights.”

#### Distancing

Distancing, which is based on participants ability to create a space between themselves and their story, was made possible specifically because filmmaking, much like other types of arts therapies, uses visual media in artistic ways, as opposed to (or in addition to) oral narration of the trauma. However, filmmaking also differs from other arts therapies which emphasize the enactment of experiences (such as drama therapy) in that it produces a concrete and permanent end-product – the film – which gives participants the opportunity to look at it repeatedly, and to gain insights, even after the intervention ends. The existence of the film also provides participants with a way to communicate their experience to others. As Solomon described:

When you make the movie, and you see your story there, you can hold it. I realized that I can put my own mirror in front of me and have a kind of resonance between me and my story. Which is rather rare, because we usually can’t tell our stories to ourselves.

Another factor which promoted distancing was the fact that each film was a joint product. Therefore, in most films, the plotline included aspects of *all* group members’ experiences, and even when there was only one actor, he/she played out aspects of the others’ stories. As such, it was easy for participants to identify with the film, and yet not feel that it was an identical representation of their own experiences; they were therefore able to avoid feeling overwhelmed by it. As Myra said:

I found that there’s a different way to express yourself, that you can express yourself without maybe, you know, being there in person, and that maybe hopefully, the film that we made, someone else with a similar experience can relate to it. The 9/11 thing, I definitely have some PTSD from that, and it’s hard, when the day comes up, to be present, and not be where you were 14 years ago.

Larry said:

I’m happy about the process; we all come from different places in the military, different branches, but now, we kind of came up with an idea and we are all gonna share a little bit about ourselves within each story. I like what we did because there’s a part of me in it, but it still feels safe with what I’ve put in it.

When such distancing (which is referred to as “externalization” in narrative therapy) occurs, participants can start processing their experiences in a new way. For example, Mark said:

It allowed me to go through the things that I went through, and see things from a different perspective. It reminds me of a jigsaw puzzle, you know, everything is all over the place, and now, we’re putting the whole thing, the pieces, together, in the editing. So, (pauses), it makes me feel part of… I haven’t felt that way (low voice) as long as I can remember.

The third sub-theme which emerged from participants’ accounts regarding the components of the model was *sense making*. This sub-theme captures the participants’ feeling that the production process was therapeutic because it enabled them to gain a better understanding of their behavior, their emotions, and themselves, as well as to feel better. Jonathan explained how the process helped him to express something which had simply been too chaotic to share in its raw form:

You can’t have someone with PTSD just sit down and talk openly about his problems, with insight and understanding. No. It’s chaos. You need the art. You need the distance. You need the music, the puppets, so that you can project yourself onto the character, or the puppet.

And Bill added an interesting insight, not previously mentioned, regarding a unique feature of this particular medium:

I think this medium helps a lot because trauma freezes us in the situation. We were in a difficult situation, it froze us (the trauma); now we have the material, we have the evidence, we can show it, we can decide who is going to see it and when. It might be my children, it might be my friends from the battalion and I may keep it to myself without being able to show it to anybody, but I know it’s MINE.

Being able to reflect on the experience, through distancing and enacting aspects of the trauma in an artistic and symbolic way, led many participants to be able to make more sense of or construct new meanings about themselves, the trauma, and/or their coping, as Tom illustrates below:

It was a way of unboxing what I was feeling and putting it on the table, dissecting it, and using different components of it in the film. Then I felt more objective about it…and I was able to put things into perspective.

Beth said:

I felt peaceful. I’m facing my fears right now, and it feels good. Not to live in that bondage. Living in bondage is more terrible than what happened with the terrorists. I’m doing this to myself. Now, I cut those ties. I just have to walk through it now, the rest of the way. Maybe I need more therapy, more classes on it, but I really think this was helpful to me.

We conclude the findings with Samantha’s words:

This trauma has really been haunting me in my worst dreams. But it felt like already on the second day of the program I started to have a voice, a voice that I didn’t have for so long. Everything in this movie is so symbolic of my life. And it really feels like I am empowered. I haven’t been empowered in so long. I’ve been holding my head down. When I came, I was ready just to be a trooper through this whole thing. I wasn’t trying to get anything out of it, just to check the box, but I feel like it’s changed my life because I get to tell how I feel, and how it feels to not have a voice and just go along with everything. It feels good.

## Discussion

Despite the availability of effective therapies for veterans coping with trauma, many veterans refrain from reaching out for help, even when they need it. Among the reasons suggested for this gap are (a) that the verbal processing of the trauma is both difficult and insufficient, due to the visual and sensual aspects of the traumatic experience, and (b) that the tendency to avoid reminders of the trauma hinders the sharing of verbal accounts (Van der Kolk, 2015). Furthermore, the growing understanding among researchers and practitioners in the field that the impact of traumatic exposure goes far beyond symptoms, and involves identity issues and existential challenges, calls for the development of more therapeutic responses. Such responses would motivate and engage veterans in therapy, through a wide range of interventions beyond just the verbal, and would address a whole host of veterans’ needs rather than just addressing their symptoms. The developing field of trauma-informed creative arts therapies, which use the sensory-based qualities of art to help individuals communicate and process traumatic memories, is testament to this growing recognition (Malchiodi, 2005, 2012; Talwar, 2007; Frydman and McLellan, 2014; Sajnani and Johnson, 2014). However, despite the promise of these therapies, they are still lagging behind in terms of providing evidence for their efficacy (Baker et al., 2017). Moreover, there is insufficient theoretical knowledge regarding what mechanisms play a therapeutic role in these therapies. The current study, although it did not directly address the question of efficacy of video-based therapy, aimed to shed light on the participants’ views regarding the curative factors that characterize this type of intervention. Three themes, which captured the participants’ experience of taking part in the program, emerged: Regaining a sense of agency, regaining a sense of belonging, and processing the trauma. Each one of these themes addresses emotions and cognitions with which many veterans cope, such as loneliness and alienation (Stein and Tuval-Mashiach, 2015a), helplessness and shattered world assumptions (Janoff-Bulman, 1992), profound changes in self- perception, and loss of personal meanings (Park, 2010). Cumulatively, these changes amount to what can be termed a “break in one’s narrative” (Tuval-Mashiach et al., 2004).

Interestingly, our findings highlight factors which are either not addressed in most standard treatments for trauma or differ significantly. Specifically, our findings indicate that to alleviate suffering, it is crucial to address the veteran’s need for a sense of belonging and being with others who are “in the same boat.” Nevertheless, this crucial aspect does not seem to figure in any part of most of the existing protocols for PTSD treatment. Our findings also point to the important role played by the reconstruction of the trauma narrative, as has been previously suggested by several researchers (Schauer et al., 2005; Robjant and Fazel, 2010; Peri and Gofman, 2014).

Our model parts ways from other models, however, on at least two grounds. Whereas some exposure therapies suggest that “a full exposure” is necessary for alleviating symptoms (Rauch et al., 2012), our intervention stipulates no such requirement. On the contrary, we would suggest that exposure should be gradual, or partial, and that the level of exposure should be freely chosen by the veteran him/herself. In addition, our findings suggest that traumatized people prefer to be actively engaged in shaping their own personal therapeutic journeys, rather than being passively guided by therapists.

Although some of the abovementioned themes are common to all or most therapeutic interventions (e.g., promoting safety, feeling understood), and some have aspects in common with arts therapies (and specifically with drama therapy, e.g., distancing, group work), we would suggest that some of the themes captured in the current study are unique to the medium of filmmaking (e.g., film editing). In what follows, we will refer to what we view as the main mechanisms which are central to the filmmaking process. Several components of the IWT video-therapy model are considered to be curative factors shared by other creative arts therapies in general. These include: collaboration, artistic expression of emotions, and using one’s imagination and creativity – all of which have been described elsewhere (e.g., Malchiodi, 2005; Orkibi, 2011). In this sense, although video-based therapy is not formally defined as one of the creative arts therapies, it shares several crucial aspects with both art therapy and drama therapy. However, we would suggest that several ideas relating to the video medium *per se*, and to the process of film production, are unique to video-therapy.

First, the *active role* of participants in the process – in defining their level of involvement, their role on the team, what and how much to share, and how to express their ideas through the film – is a central aspect of filmmaking. The instructors are in the background of the process and serve as facilitators rather than as active guides of the process. Second, the medium of filmmaking combines several *sensory-based qualities*, such as the visual, the auditory, and the verbal, as well as bodily sensations, thereby cumulatively enabling a more holistic processing of the traumatic memory. Third, filmmaking is a natural vehicle for processing the trauma narrative and integrating it into one’s life story: The process of filmmaking, much like writing a story, involves selecting between options, choosing the focus of the film, editing, and deciding on the film’s endpoint. Lastly, because the *film is a concrete product* (unlike for example a dramatic enactment in drama therapy), clients are able to go back and revisit the film, enabling them on subsequent occasions to reflect on the traumatic memory in a desensitized manner. Furthermore, the film may become a tool for fostering communication with others about difficult experiences. Given that veterans’ avoidance symptoms and difficulties in communicating with significant others about their military service constitutes one of the major obstacles to seeking therapy, the incorporation of a video-based intervention might very well serve as an engagement tool and as a first step in the veterans’ journey to healing. In sum, we believe that the use of video has great potential for veterans coping with military-related stress and trauma. In addition, although the program was developed for the purpose of working with military-related trauma, the generic model can easily be adapted to other populations coping with traumatic events, as well as to other therapeutic contexts, such as those in which people are coping with loss, illness or crisis.

The findings of this study must be considered considering several limitations. First, the veterans who did not complete the program were not included in this study; the findings therefore reflect only the experience of those participants who remained in and benefited from the program. Although there was only a 5% attrition rate, the feedback from those who did not remain could have been helpful; their input and experiences might have shed valuable light on the issue of who would be most likely to benefit from this intervention. In addition, more research should be conducted on the efficacy of the IWT filmmaking process as a therapeutic model, and on other video-based therapies in general. Future research should examine the mediating role of the curative factors found in this research. Despite these limitations, we believe that the use of filmmaking in therapy has great potential in alleviating the damage incurred by trauma and loss.

## Ethics Statement

The study was exempt from the ethics committee requirement, because this paper resulted from program evaluation and not research, *per se*. The quantitative study which assesses the intervention is approved by the Bedford VA hospital, and is carried out in accordance with the recommendations of Bedford VA IRB committee. Bedford VA IRB instructions exempt the qualitative program evaluation efforts related to this project from IRB review.

## Author Contributions

All authors are involved with preparing the manuscript for publication and involved in data collection, RT-M was responsible for qualitative research design, BP and RT-M were responsible for data analysis. CD was involved in writing the paper.

## Conflict of Interest Statement

The authors declare that the research was conducted in the absence of any commercial or financial relationships that could be construed as a potential conflict of interest.
